# Probiotic Alternative to Chlorhexidine in Periodontal Therapy: Evaluation of Clinical and Microbiological Parameters

**DOI:** 10.3390/microorganisms9010069

**Published:** 2020-12-29

**Authors:** Andrea Butera, Simone Gallo, Carolina Maiorani, Domenico Molino, Alessandro Chiesa, Camilla Preda, Francesca Esposito, Andrea Scribante

**Affiliations:** Section of Dentistry–Department of Clinical, Surgical, Diagnostic and Paediatric Sciences, University of Pavia, 27100 Pavia, Italy; andrea.butera@unipv.it (A.B.); carolina.maiorani01@universitadipavia.it (C.M.); domenicomolino160993@gmail.com (D.M.); alessandro.chiesa@unipv.it (A.C.); camilla.preda@unipv.it (C.P.); francesca.esposito@unipv.it (F.E.)

**Keywords:** dentistry, periodontitis, scaling and root planing, probiotics, chlorhexidine, periodontology, clinical trial

## Abstract

Periodontitis consists of a progressive destruction of tooth-supporting tissues. Considering that probiotics are being proposed as a support to the gold standard treatment Scaling-and-Root-Planing (SRP), this study aims to assess two new formulations (toothpaste and chewing-gum). 60 patients were randomly assigned to three domiciliary hygiene treatments: Group 1 (SRP + chlorhexidine-based toothpaste) (control), Group 2 (SRP + probiotics-based toothpaste) and Group 3 (SRP + probiotics-based toothpaste + probiotics-based chewing-gum). At baseline (T_0_) and after 3 and 6 months (T_1_–T_2_), periodontal clinical parameters were recorded, along with microbiological ones by means of a commercial kit. As to the former, no significant differences were shown at T_1_ or T_2_, neither in controls for any index, nor in the experimental groups for adherent gingiva and gingival recession. Conversely, some significant differences were found in Group 2 and 3 for the other clinical indexes tested. Considering microbiological parameters, no significant differences were detected compared to baseline values for any group, except in Group 2 and 3 at T_2_ only for the percentage of the orange complex pathogens and for the copies/microliter of *Prevotella intermedia* and *Fusobacterium nucleatum*. Accordingly, although colonization of probiotic bacteria has not been assessed in this study, the probiotics tested represent a valid support to SRP with a benefit on several clinical indexes and on specific periodontopathogens. Despite this promising action, the relationship between the use of probiotics and improvement in clinical parameters is still unclear and deserves to be further explored.

## 1. Introduction

Periodontitis is an inflammatory process affecting soft and hard tooth-supporting tissues which represents the second cause of tooth loss worldwide after dental caries [1]. This condition derives from an untreated gingivitis related to bacterial plaque accumulation and clinically causes an alteration of the marginal gum, bleeding on probing, and finally an irreversible periodontal attachment loss with formation of pockets and recessions, as well as a bone resorption with tooth mobility and exfoliation [2].

The risk factors associated with periodontitis include smoke [3], quantitative or functional reductions of polymorphonuclear leukocytes [4], immunosuppressant drugs or diseases associated with immunosuppression [5], diabetes [2] and genetic polymorphisms of genes related to the production of cytokines [6], although the accumulation of bacterial plaque remains the “*primum movens*”. In fact, biofilms with specific compositions of bacteria give rise to a periodontal inflammation which is responsible for tissue destruction in subjects with predisposing factors [7].

As regards the treatment, which aims to stop the progression of the disease, it consists of the removal of the bacterial aetiological factor. Scaling and root planing (SRP) is a non-surgical therapy aimed both to remove dental plaque and calculus (scaling) and to smooth the root surfaces infected by bacteria (root planing); these procedures have been always considered the gold standard [8]. However, the major shortcoming for SRP is the bacterial recolonization following the treatment [9]. For this reason, other therapies have been introduced as adjunctive, like the antibiotic therapy, the antimicrobial photodynamic therapy and, more recently, the probiotic therapy [10]. In particular, this last one is gaining importance thanks to the avoidance of the side-effects of antibiotics [11]. According to the definition of the Food and Agriculture Organization (FAO) and the World Health Organization (WHO), probiotics are “live microorganisms which when administered in adequate amounts confer a health benefit on the host” [12]. The mechanisms proposed to explain the beneficial action of probiotic organisms are various: exclusion and competition with pathogens for nutrients and epithelial cell adhesion, production of antimicrobial substances against pathogenetic bacteria, immunomodulation and enhancement of the mucosal barrier function [13]. Research has mainly focussed on testing the effects on periodontal disease exerted by microorganisms of the genus Lactobacillus but recently even the genus Bifidobacterium is being considered [10].

Many studies demonstrated that probiotics administered in form of lozenges are effective in potentiating the effects of SRP with a decrease of pathogens related to the disease, a recovery of periodontal clinical indexes and a reduction of proinflammatory cytokines [13,14,15,16,17]. However further randomized clinical trials should be conducted to confirm the outcomes of probiotics on both clinical and microbiological parameters, as well as to test new formulations of probiotics besides the common lozenges.

The aim of this randomized clinical trial is to analyse the adjuvant efficacy of a new probiotics-based toothpaste in addition to SRP in improving periodontal clinical indexes and microbiological parameters. Moreover, the association between the same toothpaste and a new probiotics-based chewing gum will also be tested in addition to SRP. The use of a chlorhexidine-based toothpaste following SRP will be considered as control. The first null hypothesis of the study is that there are no significant differences in clinical indexes for the groups between T0, T1 and T2. The second null hypothesis is that no differences neither occur when considering the microbiological parameters.

## 2. Materials and Methods

### 2.1. Materials 

The products used for the experimentation and their characteristics are shown in Table 1.

### 2.2. Randomized Clinical Trial 

#### 2.2.1. Trial Design

This is a parallel-group, randomized, active controlled, and single-center trial with a 1:1 allocation ratio, approved by the Unit Internal Review Board (registration number: 2019-0601)

#### 2.2.2. Participants

Patients addressing to the Unit of Dental Hygiene, Section of Dentistry, Department of Clinical, Surgical, Diagnostic and Paediatric Sciences of the University of Pavia (Pavia, Italy) were recruited from May 2019 to July 2019 and the study lasted until January 2020. The consent of participants was required. Both interventions and outcomes assessment were conducted in the abovementioned center.

The inclusion criteria were the following: age between 18–70 years, presence of periodontal disease (stage II–III), presence of single- and multiple-rooted teeth, presence of bilateral pathological probing (at least one element per side until a maximum of 20 elements in total) and good compliance of the patient. Conversely, the exclusion criteria were pregnancy, patients with cardiac stimulator or reporting neurological/psychological diseases and intake of antibiotics or anti-inflammatories in the last six months.

#### 2.2.3. Interventions and Outcomes 

At the first appointment (T_0_), after obtaining the patients’ signature as consent for the study, an instructed operator assessed the following periodontal clinical indexes by means of a probe (UNC probe 15; Hu-Friedy, Chicago, IL, USA), as reported in literature: Probing Pocket Depth (PPD) [10], Clinical Attachment Level (CAL) [10], Bleeding on Probing (BOP) [18], Bleeding Score (BS), Sulcus Bleeding Index (SBI) [19], Approximal Plaque Index (API) [20], Plaque Index (PI) [10], Adherent Gingiva (AG) (distance between the mucogingival junction and the projection on the external surface of the bottom of the gingival sulcus), Gingival Recession (GR) [10] and Pathological Sites (PS).

The five sites with the highest PPD previously assessed were isolated with cotton rolls and gentle drying with compressed air; microbiological samples were so collected with sterile paper points. For each site, one paper was inserted until the bottom of the pocket and left for 60 s. This part was conducted using the commercial BPA kit (Bacterial Periodontal Assessment, Biomolecular Diagnostic, Firenze, Italy), specific for microbiological analyses in dentistry. According to the procedures reported in the kit, resulting samples were inserted into a respective test tube and sent to the manufacturer in order to detect the percentage of total pathogens and that of pathogens belonging to the red complex (*Porphyromonas gingivalis*, *Porphyromonas endodontalis, Tannerella forsythia*, *Troponema denticola, Peptostreptococcus micros, Filifactor alocis, Synergistetes)*, enlarged red complex (*Aggregatibacter actinomycetemcomitans*), orange complex (*Prevotella intermedia*, *Fusobacterium nucleatum, Campylobacter rectus, Rothia dentocariosa, Leptotrichia hofstadii*), as well as to quantify the copies per microliter of both the total bacterial count and of the most representative pathogens abovementioned (*Tannerella forsythia, Porphyromonas gingivalis*, *Troponema denticola, Aggregatibacter actinomycetemcomitans*, *Prevotella intermedia* and *Fusobacterium nucleatum*). According to the Manufacturer’s protocol, the DNA extraction was conducted by means of QIAsymphony (QIAGEN, Hilden, Germany). Real-time polymerase chain reaction (PCR) with SYBR Green assays were performed using Rotor-Gene Q (QIAGEN) apparatus to quantify periodontopathogens [21].

Following the samples’ collection, a professional supragingival and subgingival oral hygiene was conducted using a piezoelectric (Multipiezo, Mectron S.p.a, Carasco, Italy) and Gracey curettes (Hu-Friedy, Chicago, IL, USA), and finally periodontal pockets were decontaminated using a decontaminating powder (Glycine Powder, Mectron S.p.a., Carasco, Italy).

Participants, after being motivated to oral hygiene, were subdivided into three groups according to the domiciliary treatment assigned: Group 1 was instructed to use the chlorhexidine-based toothpaste Curasept Regenerative Treatment 0.20% twice a day for six months, whereas the use of the toothpaste Biorepair Peribioma was assigned to Group 2 with the same timing. Finally, Group 3 was asked to associate the use of the same toothpaste of Group 2 with the chewing gum Biorepair Peribioma, this last used for 20 min per day, for the last 10 days of each month.

Participants underwent a 6-month follow-up with appointments at 3 months (T_1_) and 6 months (T_2_) after baseline. At each appointment, the same procedures were conducted as at baseline including assessment of periodontal clinical indexes, microbiological tests, professional supragingival and subgingival oral hygiene, periodontal pockets decontamination, as well as further motivation to oral hygiene and to the domiciliary treatment assigned.

The protocol of the study is shown in Table 2.

#### 2.2.4. Sample Size

Sample size calculation (Alpha = 0.05; Power = 90%) for three independent study groups and a continuous primary endpoint required 60 total participants of which 20 controls (9 males and 11 females, mean age 55 years old), 20 trials belonging to Group 2 (13 males and 7 females, mean age 49 years old) and 20 trials belonging to Group 3 (10 males and 10 females, mean age 55 years old). A total of 65 patients were visited before the beginning of the study, but 2 refused to participate and 3 did not meet the inclusion criteria. 60 final subjects when then selected, as requested by the sample size calculation. The following mathematical formula was used for sample size calculation:

(1) $$\mathrm{Sample}\;\mathrm{size}\;=\;\frac{Z_{\left(1-\frac{\alpha }{2}\right)}^{2}p\left(1-p\right)}{d^{2}}$$

where $z_{\left(1-\frac{\alpha }{2}\right)}$ is the standard normal variate corresponding to 1.96 at 5% type 1 error, *p* is the expected proportion in population expressed as decimal and based on previous studies [14,22], and finally *d* is the confidence level decided by the researcher and expressed as decimal too [23]. Concerning the variable Plaque index an expected mean of 61% was hypothesized, with a standard deviation of 18%. The expected difference between the means was supposed to be 0.19, therefore 20 patients were requested for each group [14].

**Figure 1:**
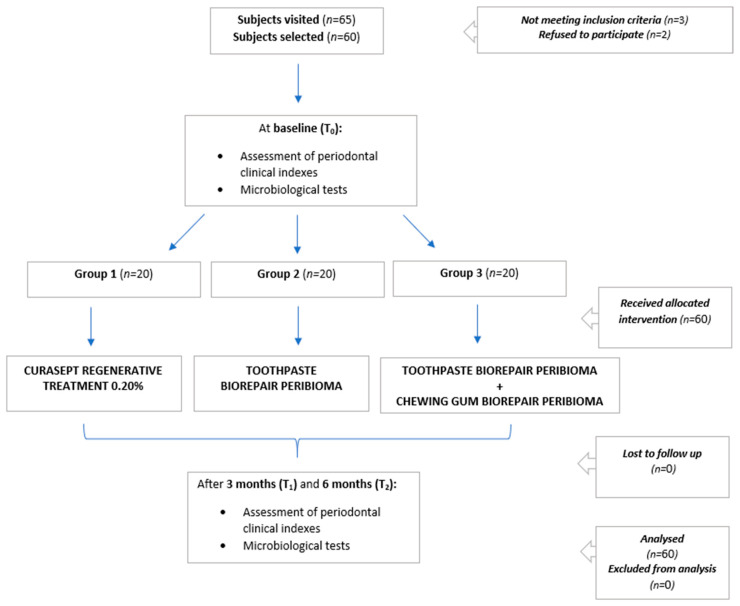
Flow-chart of the study.

The flow-chart of the study is shown in Figure 1.

#### 2.2.5. Randomization and Blinding

By means of a block randomization table, the data analyst provided a randomization sequence, considering a permuted block of 20 participants. The operator who enrolled participants also assigned them to the respective domiciliary treatment using sequentially numbered and sealed envelopes with the allocation cards previously prepared; blinding him was not technically possible. Professional oral procedures and outcomes assessment were executed by another operator. Microbiological tests were conducted by a microbiologist in an external laboratory. Operator/data assessor, microbiologist and data analyst were always blinded during the study since none of them knew the treatment administered to each participant. Patients were asked not to reveal their respective treatment to the operator/data assessor.

#### 2.2.6. Statistical Methods

Data were submitted to statistical analysis with R Software (R version 3.1.3, R Development Core Team, R Foundation for Statistical Computing, Wien, Austria).

For each group and variable, descriptive statistics (mean and standard deviation) were calculated. PPD and CAL were calculated in millimetres; BOP, API, PI, PS, Pathogen Bacteria, Enlarged Red Complex, Red Complex and Orange Complex were calculated in percentage; BS, SBI, AG and GR were calculated with the relative score; finally Total Bacteria Count as well as the presence of each specific microorganism considered were expressed as number of copies per microliter.

Data normality was calculated using the Kolmogorov–Smirnov test. For each variable, inferential comparisons among groups were performed using ANOVA with post hoc Tukey tests. Repeated measure adjustment was performed when comparing the results of the three times for each of the three different conditions tested.

Significance was predetermined for *p* < 0.05 for all the tests performed.

## 3. Results

The results of the study are shown in Table 3, Table 4 and Table 5.

### 3.1. Periodontal Parameters

#### 3.1.1. Probing Pocket Depth (PPD) and Clinical Attachment Level (CAL)

**Figure 2:**
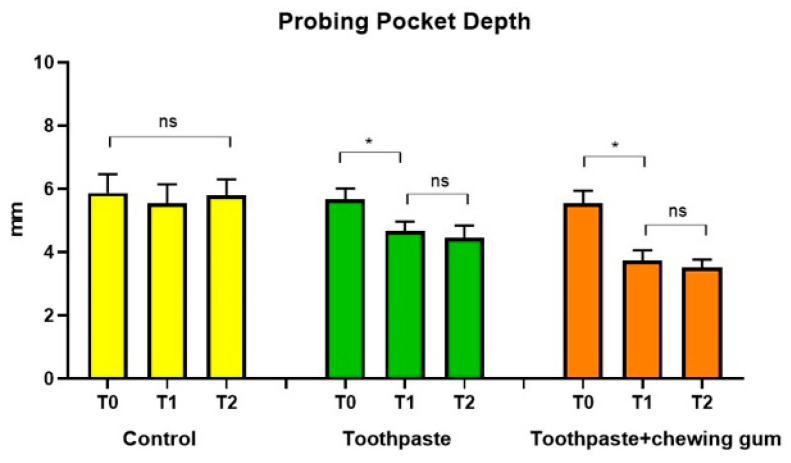

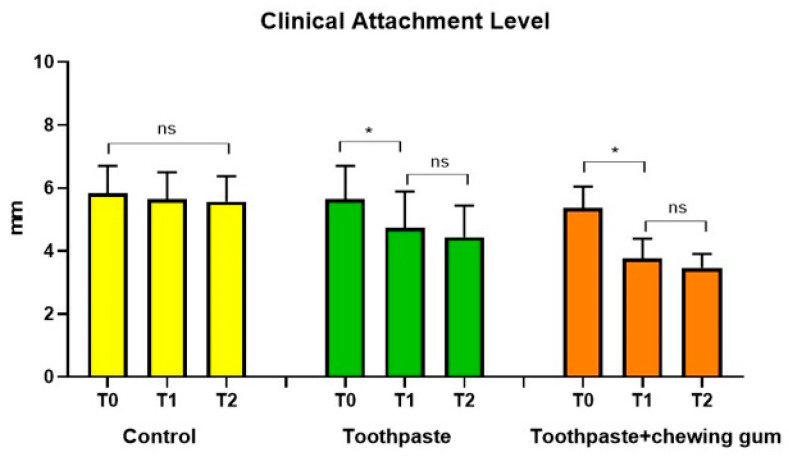
Probing Pocket Depth (mm); Clinical Attachment Level (mm)**.** *: statistically significant difference (*p* < 0.05); ns: not significant difference (*p* > 0.05).

No significant differences were found in Group 1 (control) at any endpoint. For both Group 2 (toothpaste) and Group 3 (toothpaste + chewing gum), a significant difference was found between T_0_ and T_1_, but not between T_1_ and T_2_ (Figure 2).

#### 3.1.2. Bleeding on Probing (BOP), Bleeding Score (BS), Sulcus Bleeding Index (SBI), Approximal Plaque Index (API), Plaque Index (PI) and Pathological Sites (PS)

**Figure 3:**
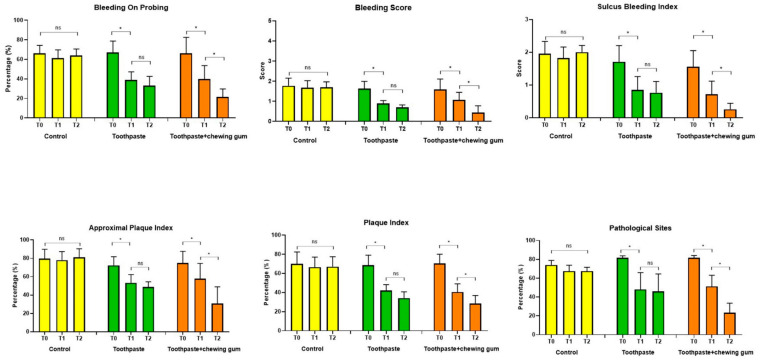
Bleeding On Probing (%); Bleeding Score (0–5); Sulcus Bleeding Index (0–3); Approximal Plaque Index (%); Plaque Index (%); Pathological Sites (%). *: statistically significant difference (*p* < 0.05); ns: not significant difference (*p* > 0.05).

No significant differences were found in Group 1 (control) at any endpoint. For both Group 2 (toothpaste) and Group 3 (toothpaste + chewing gum), a significant difference was found between T_0_ and T_1_, whereas the difference between T_1_ and T_2_ was statistically significant only for Group 3 (Figure 3).

#### 3.1.3. Adherent Gingiva (AG) and Gingival Recession (GR)

**Figure 4:**
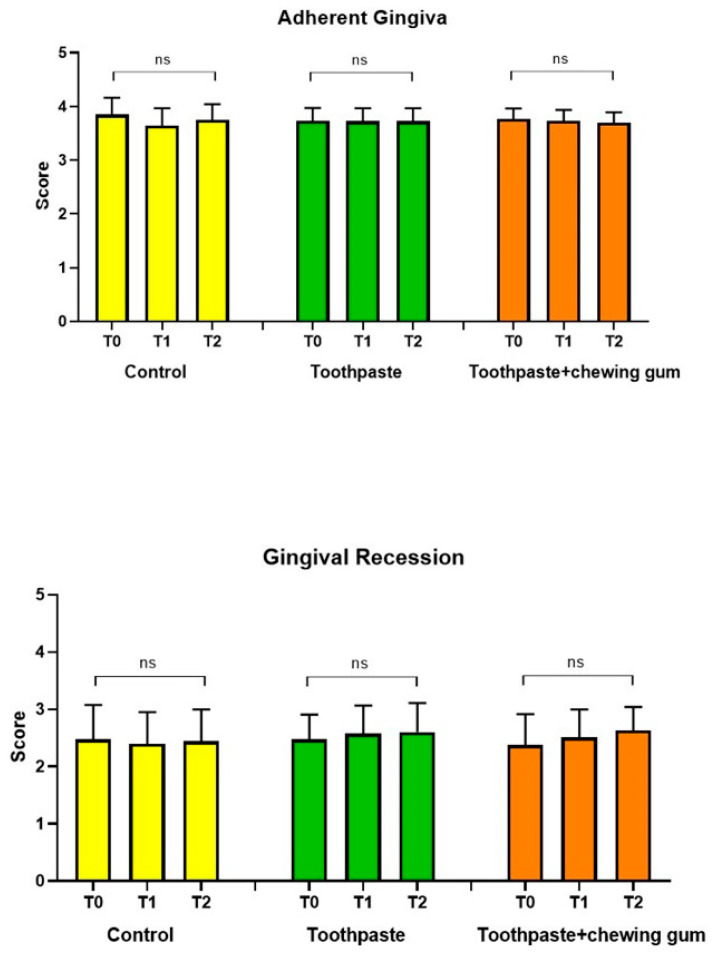
Adherent Gingiva (0–5); Gingival Recession (0–5)**.** ns: not significant difference (*p* > 0.05).

No significant differences were found at any endpoint, independently of the group considered (Figure 4).

### 3.2. Microbiological Parameters

#### 3.2.1. Pathogen Bacteria, Enlarged Red Complex, Red Complex, Total Bacteria Count, *Aggregatibacter Actinomycetemcomitans*, *Tannerella Forsythia*, *Porphyromonas Gingivalis* and *Troponema Denticola*

**Figure 5:**
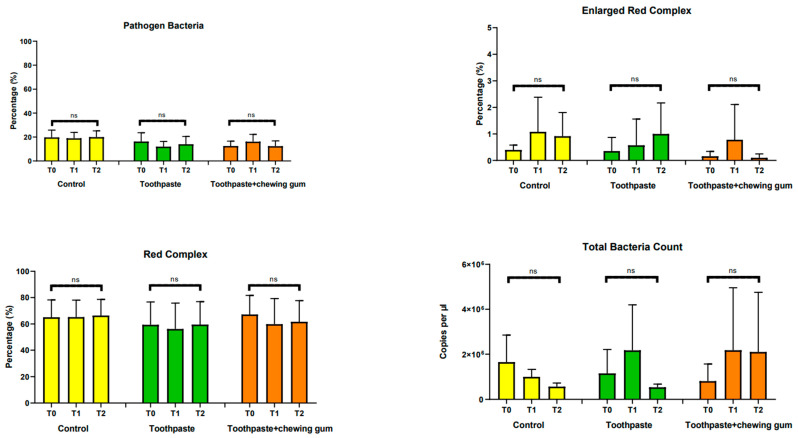

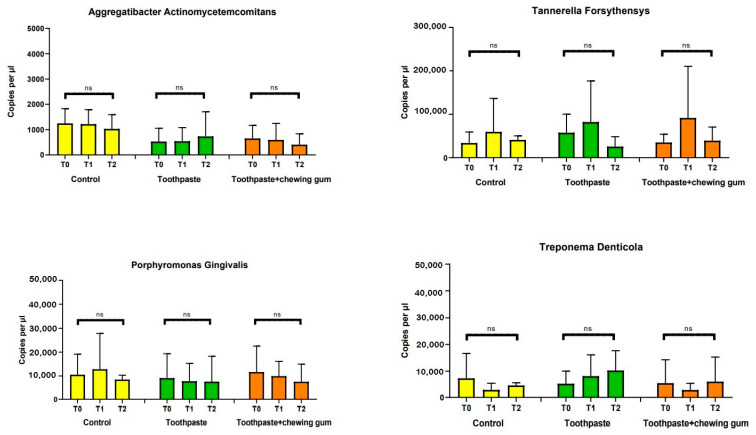
Pathogen Bacteria (%); Enlarged Red Complex (%); Red Complex (%); Total Bacteria Count (copies/µL); *Aggregatibacter actinomycetemcomitans* (copies/µL); *Tannerella forsythia* (copies/µL); *Porphyromonas gingivalis* (copies/µL); *Troponema denticola* (copies/µL). ns: not significant difference (*p* > 0.05).

No significant differences were found at any endpoint, independently of the group considered (Figure 5).

#### 3.2.2. Orange Complex, *Prevotella Intermedia* and *Fusobacterium Nucleatum*

**Figure 6:**
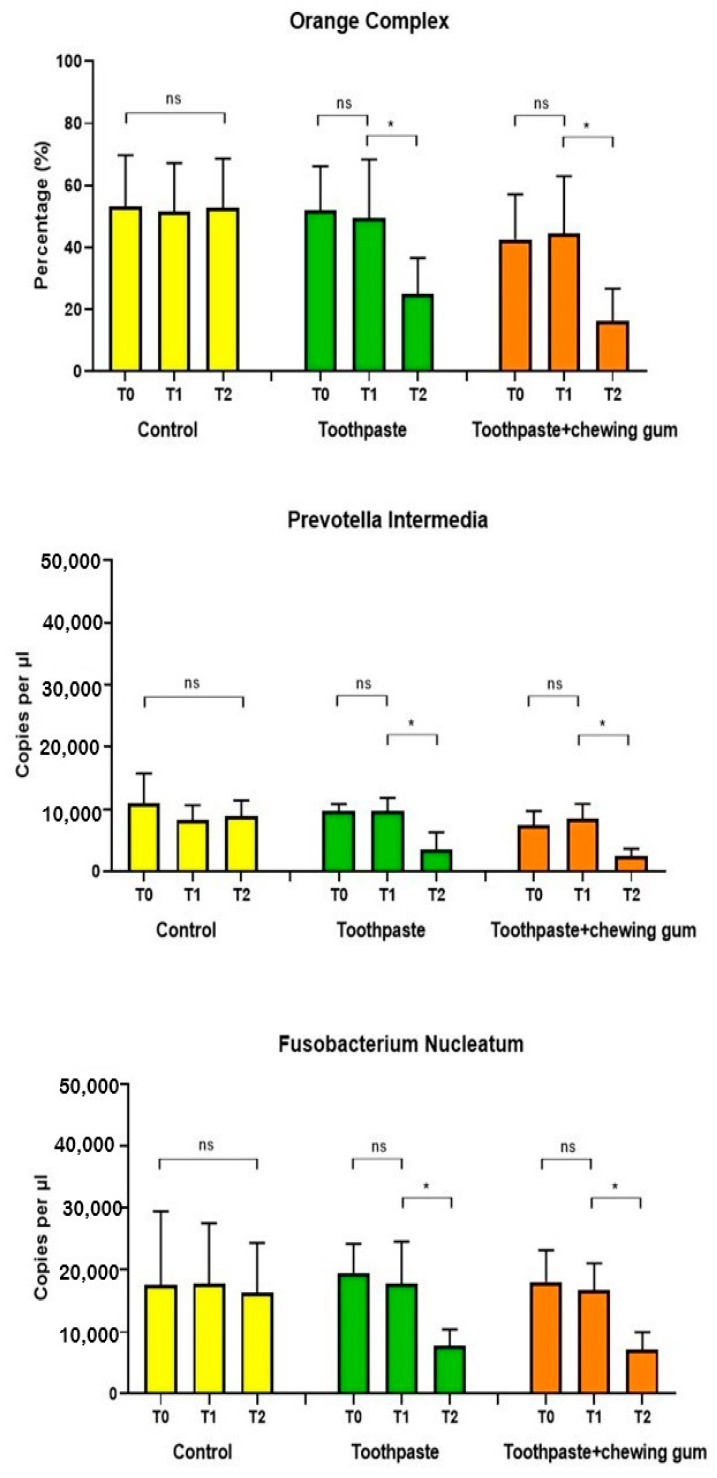
Orange Complex (%); *Prevotella intermedia* (copies/µL); *Fusobacterium nucleatum* (copies/µL). *: statistically significant difference (*p* < 0.05); ns: not significant difference (*p* > 0.05).

No significant differences were found in Group 1 (control) at any endpoint. For both Group 2 (toothpaste) and Group 3 (toothpaste + chewing gum), no significant difference was found between T_0_ and T_1_, but only between T_1_ and T_2_ (Figure 6).

## 4. Discussion

Oral infections represent an important concern in dentistry and many efforts are being made to contrast bacterial colonization [24,25]. In addition to infection of the teeth by cariogenic bacteria resulting in both primary [26] and secondary [27] decay, even tooth-supporting tissues can be jeopardized in susceptible patients by pathogenic microorganisms causing a dysbiosis leading to periodontitis [28].

Despite SRP is still the gold standard treatment for periodontitis, many limitations, like bacterial recolonization, are associated with this therapy; among the adjunctive strategies proposed, the use of probiotics is more and more recommended, considering the avoidance of antibiotics’ side effects [29]. The rational of the administration of symbiotic bacteria consists of a change of the local environment allowing SRP to resolve inflammation and prevent further recolonization [10].

To date, different Authors have evaluated the efficacy of probiotics of the genus *Lactobacillus*, especially administered in form of lozenges, as adjunct to non-surgical periodontal treatment in periodontitis. Conversely, in the present study, new formulations of probiotics recently commercialized have been tested, including both a toothpaste and a chewing gum. Additionally, the former product also contains probiotics of the genus *Bifidobacterium*, which has been still poor tested so far.

The first null hypothesis of the study was partially rejected. Whereas the chlorhexidine-based toothpaste considered as control hasn’t exert a significant effect for any periodontal variable, probiotics have shown an effect on periodontal parameters and this was different according to the only use of the experimental toothpaste or the association of this one with the experimental chewing gum. As expected, no difference was shown for AG and GR at any endpoint for any group; conversely, when considering PPD and CAL, a significant reduction of the probing as well as of the clinical attachment loss was obtained after 3 months of domiciliary hygiene with the probiotics-based toothpaste and this effect was even higher if associating the use of the chewing gum for 20 min per day for the last 10 day of each month. No further significant difference was assessed during the 6-month follow up. A similar result was noticed for BOP, BS, SBI, API, PI and PS, but in these cases the adjunct of the chewing gum was effective in improving periodontal parameters even between the third and the sixth month.

According to these results, the toothpaste containing probiotics of the genus *Lactobacillus* and *Bifidobacterium* has been a valid support following SRP in improving periodontitis. In recent years, different studies have been conducted to test the effect of probiotics of the genus *Lactobacillus* in periodontology. For example, Ikram et al. [30] have compared the clinical efficacy of the local probiotic *Lactobacillus reuteri (L. reuteri)* (in form of powder mixed in water and applied with a toothbrush around gingival margins for 5 min twice a day) with systemic amoxicillin 500 mg, in addition to SRP. A similar improvement for all clinical periodontal parameters was found for both treatments. Anyway, as previously reported, probiotics are thought to overcome the limitations associated with the antibiotic therapy. Other studies confirm the benefits of *L. reuteri* with respect to SRP alone, and this is related to a reduction of pro-inflammatory cytokines and periodontal pathogenic bacteria [16,17,31].

However, in the present study, the experimental toothpaste contained not only bacteria of the genus *Lactobacillus* but also *Bifidobacterium*, despite the specific strains of these bacteria do not appear in the statement of the Manufacturer. So far, few studies have been conducted to test even these microorganisms. One of the first randomized clinical trial was conducted by Invernici et al. [10] who demonstrated that the use of *B. lactis* HN019 in form of lozenges following SRP promotes additional clinical, microbiological, and immunological benefits in the treatment of chronic periodontitis; therefore, our results agree with this study and it might be assumed that the combination of *Lactobacillus* and *Bifidobacterium* of the experimental toothpaste tested might exert a synergic effect.

Moreover, when the experimental chewing gum was associated with the abovementioned toothpaste, a further effect was seen after 6 months of follow up for bleeding- and plaque-related indexes, but not for PPD and CAL. This additional effect might be related to the specific probiotics’ composition of the chewing gum which contains, according to the Manufacturers, the microorganisms *L. reuteri* (SGL 01), *L. salivarius* (SGL 03) and *L. plantarum* (SGL 07).

The second null hypothesis was partially rejected. Independently of the control or experimental groups, no significant differences were found at any endpoint neither for the percentage of pathogen bacteria, enlarged red complex and red complex, neither for the number of copies per microliter of total bacteria, *Aggregatibacter actinomycetemcomitans, Tannerella forsythia*, *Porphyromonas gingivalis* and *Troponema denticola*. As well, no differences were shown in the control group as regards the percentage of the orange complex and the number of copies per microliter of *Prevotella intermedia* and *Fusobacterium nucleatum*, whereas for group 2 and 3, respectively assigned to the use of the probiotics-based toothpaste and to the association of this last one with the experimental chewing gum, these three variables were significantly decreased but only between the third and sixth month of follow up. According to these data, the experimental treatments tested in this study didn’t influence neither the percentage of pathogen bacteria nor the quantity of those pathogens more strictly related to periodontitis. However, both the use of the probiotics-based toothpaste as well as its association with the probiotics-based chewing gum have similarly reduced the number of copies per microliter of pathogens belonging to the orange complex, these last again related to periodontitis even though with a minor risk if compared to pathogens of the red complex. It is interesting to notice that, independent of the specific experimental treatment (and so of the genus of probiotics), the positive effect of probiotics on microbiological parameters has become significant only after the third month of treatment, whereas periodontal clinical indexes generally improved in a significant way even during the first three months. Based on this consideration, as well as on the fact that no variation regarded periodontal pathogens belonging to red and enlarged red complex, the relationship between the use of probiotics and the improvement in clinical parameters is still unclear. It might be supposed that the improvement following the probiotic-based therapy might be related not only to a reduction of microbial counts but, above all, to a switch from a pro-inflammatory response towards an anti-inflammatory one. This possibility completely agrees with the concept of periodontitis as the result of the tissues’ destruction exerted by inflammatory/immune cells.

According to the previously mentioned study of Invernici et al. [10] which compared the effect of *B. lactis* HN019 in form of lozenges as adjuvant to SRP with respect to SRP + placebo, the additional administration of probiotics was effective in reducing the mean proportions of orange complex at 30 days as well as that of red complex at 90 days. Despite this is not in accordance with our results, it may be assumed that the different outcome might be related to a different clinical situation of the patients at baseline. Moreover, in case of a longer follow up for our study, a reduction of bacteria belonging to the red complex might have occurred following that of orange complex, as assessed by the previous Authors.

Considering the association SRP + chlorhexidine-based toothpaste or SRP + probiotics (in form of chewing gum and/or toothpaste), our results have shown, with surprise, no influence at all by the first treatment neither on clinical nor on microbiological parameters. This might seem strange considering that SRP is regarded as the gold standard for treating periodontitis but could be explained since participants had already been treated and a significant improvement following SRP had been obtained previously. Few studies have compared the use of chlorhexidine and probiotics: for instance, these substances have been tested in different formulations and with different purposes such as antimicrobics and plaque inhibitor in orthodontic patients [32], as well as for the treatment of peri-implant mucositis [33]. Due to the heterogeneity of the studies, no comparisons can be conducted with our results. In addition to that, to the best of our knowledge, this seems to be the first randomized clinical trial testing probiotics in form of toothpaste and chewing gum (instead of mouthwash or lozenges) as a support to the traditional SRP, and to conduct a comparison with the combination SRP + chlorhexidine. Therefore, these preliminaries results should be confirmed by further clinical trials.

The main limitation of the present report is that no data have been assessed regarding the effective colonization of periodontium by probiotic bacteria, but the only reduction of periodontal pathogens has been measured. Other limitations can be related both to participants, considering an eventual low compliance of some of them, but also to the operator assessing the clinical data since no calibration values, Kappa indices or intra-inter correlation agreement values were considered. In addition to that, the first examination after baseline was conducted three months later; this timing could be considered not properly adequate because an assessment after 4–6 weeks is generally required following SRP since this time corresponds to the period necessary for gingival tissues to mature and heal. Additionally, the overall follow up lasting until the sixth month of treatment could be considered relatively short. As regards this last point, the risk of a long-term bacterial recolonization still represents the major concern of periodontal therapy. Accordingly, next studies should be realized considering a longer follow up, in order to assess not only the superiority of probiotics to the traditional chlorhexidine, but also a major efficacy in preventing bacterial recolonization of the periodontal pockets treated.

In the perspective of better understanding the relationship between the use of probiotics and the improvement of clinical parameters, further studies should focus not only on the action of the former on periodontal pathogens, but especially on the effects exerted towards human inflammatory/immune cells, considering the key role of these latter on the development of periodontitis.

## 5. Conclusions

Probiotics are representing a breakthrough for the treatment of periodontitis, also because of the absence of the eventual side effects reported after a prolonged use of chlorhexidine. Considering the clinical relevance, the new probiotics-based toothpaste and chewing gum tested in this study seem to be a valid support to SRP, with a general improvement on clinical indexes and reduction of periodontopathogens of the orange complex.

## Figures and Tables

**Table 1:** List of the products used in the study.

Product	Description	Ingredients	Manufacturer	Code
Biorepair Peribioma	Toothpaste	Aqua, Zinc Hydroxyapatite *, Sorbitol, Glycerin, Hydrated Silica, Silica, Cocamidopropyl Betaine, Cellulose Gum, Aroma, Pistacia Lentiscus (Mastic) Gum Oil, Ascorbic Acid, Tocopheryl Acetate, Retynil Palmitate, Sodium Hyaluronate, Hamamelis Virginiana Leaf Extract, Spirulina Platensis Extract, Calendula Officinalis Flower Extract, Eucaliptus Globulus Leaf Oil, Bifidobacterium *, Lactobacillus *, Sodium Myristoyl Sarcosinate, Sodium Methyl Cocoyl Taurate, Phenoxyethanol, Benzyl Alcohol, Sodium Benzoate, Sodium Saccharin, Potassium Sorbate, Maltodextrin, Citric Acid, Helianthus Annuus Seed Oil, BHT, Limonene, Eugenol, CI 77891, CI 73360. * microRepair^®^BIOMA	Coswell SPA, 40050 Funo di Argelato, Bologna, Italy	GA1504900
Biorepair Peribioma	Chewing gum	Gum base (aroma; emulsifier: soya lecithin; sweetener: acesulfame, sucralose; antioxidant: tocopherols); bulking agents: isomalt, sorbitol; microRepair^®^ (calcium salts of orthophosphoric acid) *; Probiotics * [L. reuteri (SGL 01), L. salivarius (SGL 03), L. plantarum (SGL 07), support: corn maltodextrin, anti-caking agent: silicium dioxide]; Aroma; Vitamin C (ascorbate calcium); colorant foods (radish concentrate and sweet potato); sweeteners: sucralose, acesulfame K; Vitamin D (Cholecalciferol). * microRepair^®^BIOMA	Coswell SPA, 40050 Funo di Argelato, Bologna, Italy	GA1536200
Curasept Regenerative Treatment 0.20%	Toothpaste	Purified Water, Sorbitol, Hydrated Silica, PEG-32, Cocamidopropyl Betaine, Xylitol, Cellulose Gum, Aroma, Sodium Hyaluronate, Ascorbic Acid, Chlorhexidine, Digluconate, Sodium Metabisulfite, Sodium Citrate, Titanium Dioxide (C.I. 77891), Sodium Benzoate, Sodium Saccharin, Citric Acid, C.I. 17200, C.I. 42090.	Curasept SPA, 21047 Saronno, Varese, Italy	190011661

* As stated by the Manufacturers.

**Table 2:** Protocol of the study.

Appointment	Procedures
**Baseline (T_0_)**	Signature to the informed consent for the study Assessment of periodontal clinical indexes Microbiological tests Professional supragingival and subgingival oral hygiene with piezoelectric and Gracey curettes Periodontal pockets decontamination with Air-flow Plus Motivation to oral hygiene and instruction for the domiciliary treatment: **Group 1**: toothpaste Curasept Regenerative Treatment 0.20% **Group 2**: toothpaste Biorepair Peribioma **Group 3**: toothpaste Biorepair Peribioma + chewing gum Biorepair Peribioma
**After 3 months (T_1_)** **After 6 months (T_2_)**	Reassessment of periodontal clinical indexes Microbiological tests Professional supragingival and subgingival oral hygiene with piezoelectric and Gracey curettes Periodontal pockets decontamination with Air-flow Plus Further motivation to oral hygiene and continuation of the domiciliary treatment assigned

**Table 3:** Descriptive statistics of periodontal clinical parameters.

Group	Time	PPD	CAL	BOP	BS	SBI	API	PI	AG	GR	PS
		Mean	Mean	Mean	Mean	Mean	Mean	Mean	Mean	Mean	Mean
		(SD)	(SD)	(SD)	(SD)	(SD)	(SD)	(SD)	(SD)	(SD)	(SD)
**Control**	T_0_	5.88 (1.26) ^a^	5.83 (1.87) ^a^	66.25 (17.23) ^a^	1.78 (0.80) ^a^	1.96 (0.80) ^a^	79.75 (21.61) ^a^	70.00 (26.56) ^a^	3.85 (0.67) ^a^	2.48 (1.28) ^a^	74.00 (10.46) ^a^
	T_1_	5.55 (1.27) ^a^	5.66 (1.80) ^a^	61.25 (18.16) ^a^	1.66 (0.75) ^a^	1.82 (0.73) ^a^	78.00 (20.03) ^a^	66.50 (22.72) ^a^	3.65 (0.68) ^a^	2.40 (1.18) ^a^	67.50 (13.33) ^a^
	T_2_	5.80 (1.08) ^a^	5.57 (1.72) ^a^	64.00 (14.01) ^a^	1.70 (0.57) ^a^	2.00 (0.44) ^a^	81.00 (20.17) ^a^	67.00 (22.33) ^a^	3.75 (0.62) ^a^	2.44 (1.19) ^a^	67.50 (8.66) ^a^
**Toothpaste**	T_0_	5.67 (0.74) ^a^	5.64 (2.27) ^a^	67.00 (24.94) ^a^	1.64 (0.76) ^a^	1.71 (1.06) ^a^	72.25 (20.42) ^a^	68,50 (22.48) ^a^	3.73 (0.51) ^a^	2.46 (0.92) ^a^	81.50 (4.89) ^a^
	T_1_	4.67 (0.64) ^b^	4.74 (2.45) ^b^	39.00 (17.59) ^b^	0.89 (0.32) ^b^	0.85 (0.87) ^b^	53.25 (19.42) ^b^	42.25 (12.82) ^b^	3.73 (0.52) ^a^	2.57 (1.06) ^a^	48.00 (38.06) ^b^
	T_2_	4.46 (0.84) ^b^	4.44 (2.14) ^b^	33.00 (20.39) ^b^	0.70 (0.26) ^b^	0.76 (0.74) ^b^	48.75 (12.13) ^b^	34.15 (14.08) ^b^	3.72 (0.52) ^a^	2.60 (1.08) ^a^	46.00 (39.52) ^b^
**Toothpaste + Chewing Gum**	T_0_	5.57 (0.85) ^a^	5.36 (1.46) ^a^	66.15 (34.89) ^a^	1.59 (1.10) ^a^	1.56 (1.05) ^a^	74.83 (27.38) ^a^	70.50 (20.38) ^a^	3.76 (0.43) ^a^	2.38 (1.14) ^a^	81.75 (4.94) ^a^
	T_1_	3.74 (0.69) ^c^	3.76 (1.35) ^c^	39.90 (29.23) ^b^	1.06 (0.82) ^b^	0.71 (0.87) ^b^	57.75 (35.67) ^b^	40.50 (18.20) ^b^	3.74 (0.43) ^a^	2.51 (1.05) ^a^	51.30 (25.10) ^b^
	T_2_	3.52 (0.53) ^c^	3.46 (0.94) ^c^	21.50 (17.55) ^c^	0.44 (0.72) ^c^	0.26 (0.40) ^c^	30.75 (39.01) ^c^	28.50 (17.85) ^c^	3.69 (0.42) ^a^	2.63 (0.87) ^a^	23.40 (19.48) ^c^

For each variable tested, groups with the same superscript letter (a, b or c) showed no significantly different means.

**Table 4:** Descriptive statistics of microbiological parameters (1).

Group	Time	Total Bacteria Count	AAE	PG	TF	TD	PI	FN
		Mean	Mean	Mean	Mean	Mean	Mean	Mean
		(SD)	(SD)	(SD)	(SD)	(SD)	(SD)	(SD)
**Control**	T_0_	1648650	1247.48	10530.55	34012.51	7339.82	11018	17607.3
		(2571189.00) ^a^	(1238.52) ^a^	(18424.41) ^a^	(54134.03) ^a^	(19922.95) ^a^	(10208.32) ^a^	(25342.18) ^a^
	T_1_	999745.2	1216.92	12836.1	59654.57	2973.42	8326	17834.3
		(705812.30) ^a^	(1219.51) ^a^	(32125.70) ^a^	(164197.50) ^a^	(5358.644) ^a^	(5063.76) ^a^	(20783.60) ^a^
	T_2_	561150	1030.08	8479	40990	4651	8830	16298.82
		(349477.80) ^a^	(1202.40) ^a^	(3974.44) ^a^	(19938.19) ^a^	(2158.87) ^a^	(5617.44) ^a^	(17212.96) ^a^
**Toothpaste**	T_0_	1150665	528	9107.65	57690.4	5318	9720.1	19381.2
		(2270115.00) ^a^	(1121.42) ^a^	(21882.53) ^a^	(90873,28) ^a^	(10086.63) ^a^	(2405.66) ^a^	(10360.37) ^a^
	T_1_	2173295	540.75	7780	81998.28	8153	9652.2	17719.25
		(4325099.00) ^a^	(1151.19) ^a^	(16100.69) ^a^	(202365.80) ^a^	(16980.35) ^a^	(4670.72) ^a^	(14680.71) ^a^
	T_2_	535470	734.5	7625.75	25656.4	10244.63	3536	7843.6
		(306466.90) ^a^	(2076.09) ^a^	(22714.00) ^a^	(48302.74) ^a^	(15860.15) ^a^	(5931.72) ^b^	(5509.43) ^b^
**Toothpaste + Chewing Gum**	T_0_	808115	650.25	11644.1	35091.9	5521.75	7476.55	18053
		(1619913.00) ^a^	(1114.43) ^a^	(23306.05) ^a^	(40463.06) ^a^	(18720.46) ^a^	(4787.82) ^a^	(10931.75) ^a^
	T_1_	2171589	595.95	9939.8	91622.76	2942.15	8406	16734.2
		(5938379.00) ^a^	(1387.69) ^a^	(13193.90) ^a^	(253652.70) ^a^	(5375.99) ^a^	(5333.22) ^a^	(9249.37) ^a^
	T_2_	2097731	406.2	7553.641	3929080	6065.13	2520.5	7211.78
		(5655579.00) ^a^	(919.53) ^a^	(15781.77) ^a^	(66828.91) ^a^	(19738.92) ^a^	(2435.87) ^b^	(5971.25) ^b^

For each variable tested, groups with the same superscript letter (a or b) showed no significantly different means.

**Table 5:** Descriptive statistics of microbiological parameters (2).

Group	Time	Pathogen Bacteria	Enlarged Red Complex	Red Complex	Orange Complex
		Mean	Mean	Mean	Mean
		(SD)	(SD)	(SD)	(SD)
**Control**	T_0_	19.71	0.4	65.08	53.27
		(12.93) ^a^	(0.39) ^a^	(28.13) ^a^	(35.14) ^a^
	T_1_	18.92	1.08	65.19	51.38
		(10.64) ^a^	(2.79) ^a^	(27.52) ^a^	(33.83) ^a^
	T_2_	19.90	0.91	66.38	52.69
		(11.20) ^a^	(1.91) ^a^	(26.10) ^a^	(34.08) ^a^
**Toothpaste**	T_0_	16.23	0.35	59.32	51.88
		(15.75) ^a^	(1.10) ^a^	(37.05) ^a^	(30.52) ^a^
	T_1_	11.97	0.57	56.19	49.39
		(9.22) ^a^	(2.11) ^a^	(41.82) ^a^	(40.66) ^a^
	T_2_	13.99	1	59.51	24.87
		(13.79) ^a^	(2.49) ^a^	(37.14) ^a^	(24.99) ^b^
**Toothpaste + Chewing Gum**	T_0_	12.49	0.16	67.19	42.58
		(8.73) ^a^	(0.39) ^a^	(30.91) ^a^	(31.07) ^a^
	T_1_	16.12	0.78	59.8	44.42
		(13.10) ^a^	(2.85) ^a^	(41.47) ^a^	(39.57) ^a^
	T_2_	12.31	0.1	61.66	16.34
		(9.50) ^a^	(0.31) ^a^	(34.32) ^a^	(22.13) ^b^

For each variable tested, groups with the same superscript letter (a or b) showed no significantly different means.

## Data Availability

All data are available upon request to corresponding Authors.
